# The Functionalization of a Honeycomb Polystyrene Pattern by Excimer Treatment in Liquid

**DOI:** 10.3390/polym14224944

**Published:** 2022-11-16

**Authors:** Petr Slepička, Jakub Siegel, Miroslav Šlouf, Dominik Fajstavr, Klára Fajstavrová, Zdeňka Kolská, Václav Švorčík

**Affiliations:** 1 Department of Solid State Engineering, University of Chemistry and Technology Prague, Technická 5, 16628 Prague, Czech Republic; 2 Innovation Centre of the Institute of Macromolecular Chemistry, Academy of Sciences of Czech Republic, Prague 6 Brevnov, 16200 Prague, Czech Republic; 3 Faculty of Science, J. E. Purkyne University, 40001 Usti Nad Labem, Czech Republic

**Keywords:** honeycomb, polystyrene, nanostructure, gold nanocluster, immobilization, morphology, excimer laser

## Abstract

In this article, we present a unique combination of techniques focusing on the immobilization of noble metal nanoparticles into a honeycomb polystyrene pattern prepared with the improved phase-separation technique. The procedure consists of two main steps: the preparation of the honeycomb pattern (HCP) on a perfluoroethylenepropylene substrate (FEP), followed by an immobilization procedure realized by the honeycomb pattern’s exposure to an excimer laser in a noble metal nanoparticle solution. The surface physico-chemical properties, mainly the surface morphology and chemistry, are characterized in detail in the study. The two-step procedure represents the unique architecture of the surface immobilization process, which reveals a wide range of potential applications, mainly in tissue engineering, but also as substrates for analytical use.

## 1. Introduction

Honeycomb structures have been thoroughly studied in last decade, mainly for the simplicity of their preparation based on two basic approaches, the breath figure (BF) method [1,2] and improved phase separation (IPS), developed by Bui et al. [3,4,5]. The advantage of the IPS approach relies on the fact that relatively large areas can be processed in a short period of time, with the honeycomb pattern’s homogeneity being comparable to the BF technique [6]. Applications of honeycomb structures in the research area can be mostly observed in tissue engineering practices as substrates for different types of cells [7], where the materials used for honeycomb formation may consist of biopolymers, such as Poly-l-lactic acid (PLLA) [8,9], cellulose acetate [10], or other materials [11], which also creates a wide range of possibilities for further applications, e.g., as antibacterial substrates, if noble metals, such as silver, are additively used [12]. Subsequent pattern formation can be realized by the immobilization process, as has been demonstrated for PEN (polyethylene naphthalate) in [13], the LIPSS (laser-induced periodic surface structure) pattern having an outstanding application for targeted cell growth [14,15].

Honeycombs are widely applied in the fields of electronics and sensors. The fabrication of the selective assembly of Ag nanoparticles on honeycomb films and their highly sensitive surface-enhanced Raman scattering (SERS) of R6G (rhodamine 6G) molecules were demonstrated by Zhang et al. [16]. Honeycomb films prepared based on an amphiphilic block polymer (polystyrene-block-polyacrylic acid) by the breath figure method decorated with AgNPs exhibited strong SERS of R6G molecules. The combination of parylene deposition with the polymer auto-organization phenomenon creates the breath figures mechanism. This mechanism creates stable porous films that can be filled with a chloroform solution of fluorescent materials [17]. It has been demonstrated that liquid crystals can be encapsulated inside the patterned surface between two layers of parylene, presenting interesting organization features dictated by spatial constraints, suggesting a new approach for the development of honeycomb-based liquid-crystal cells for flexible displays [18]. Triboelectric nanogenerators (TENGs) composed of PLA (polylactic acid) biomaterial, a surfactant-free graphene oxide-polylactic acid (GO/PLA) nanocomposite with customizable honeycomb patterns, was prepared via a scalable two-step solution method to achieve a power-boosted biocompatible TENG for application in healthcare areas thanks to its boosted output performance and persisting biocompatibility [19]. Additionally, exotic forms of polymers were prepared by breath figure methods, and microporous films with tunable honeycomb structures were fabricated based on the self-assembly of a series of clusto-supramolecular star polymers (CSPs) guided by breath figure templates. The CSPs consisted of a hydrophilic polyoxometalate cluster core and several hydrophobic polystyrene arms that were electrostatically connected to the core [20], which provided a facile way to incorporate inorganic functional components into microporous polymer films. As stated above, different types of incorporated particles were applied as a precursor for subsequent doped honeycomb growth. Highly ordered honeycomb films were produced from arborescent graft polystyrene (AGPS) solutions using breath figures (BF), multiwall carbon nanotubes (MWCNTs) were introduced into AGPS solutions, and the application of the BF process using the solution blends yielded patterned hybrid films [21]. A summary of the application of breath figure methods in the last 10 years and a discussion of the influencing factors of breathing figure arrays have been comprehensively described in [22]. The advantage of the direct exposure of samples in the liquid medium, such as a solution of nanoparticles [23], is an easy method for the preparation of the immobilized surface, which can be considered as a polymer–nanoparticle composite [24]; only the surface layer is affected due to the application of an excimer laser wavelength. The noble metal nanoparticles diffuse into the polymer and the isolated character of the noble metal is preserved [24]. The use of an aromatic polymer as a material for a honeycomb primary pattern presents various possibilities to combine these two techniques. Another study [25] presented a way to prepare the noble metal nanoparticles’ immobilization on self-assembled honeycomb-patterned films as a substrate suitable for surface-enhanced Raman scattering (SERS); the honeycomb pattern immobilized with noble nanoparticles or rGO was also studied in [26,27]. The creation of an LIPSS pattern on an aromatic polystyrene polymer following laser irradiation is a well-known phenomenon [28]. An LIPSS pattern created on composite aromatic polymers containing acetylsalicylic acid has also been reported in the literature [29] for polystyrene, or for different polymers [30,31].

The main topic of this paper concerns the laser irradiation of a polystyrene honeycomb construction prepared using the improved phase-separation technique in solution followed by noble metal immobilization. To the best of our knowledge, the improved phase-separation technique used for the polystyrene honeycomb construction followed by noble metal immobilization from the solution has never been used to date. Moreover, this approach can be applied to other types of aromatic polymers suitable for the construction of honeycombs by the phase-separation technique. The advantage of polystyrene is that it has been proved to be a biocompatible polymer, even as a tissue engineering standard (Petri dishes). Since it has an aromatic core in its chain, it is possible to prepare an LIPSS pattern on pristine polystyrene, or the pattern can be enhanced by noble nanoparticle immobilization if a 248 nm wavelength is applied. The assistance of a laser beam led to immobilization on the surface of the polymer’s upper layers combined with a pattern morphological update. The combination of the technique of improved phase separation followed by time-effective laser treatment is used here for the first time, to the best of our knowledge, and strongly contributes to the field of material and polymer science with possible applications in both antibacterial surfaces, constructs for soft and hard tissue engineering and sensor analysis.

## 2. Materials and Methods

### 2.1. Materials and Treatment

The perfluoroethylene propylene (FEP) polymer (the density of 2.15 g cm^−3^, 50 μm thick foils), supplied by Goodfellow Ltd. Goodfellow, Ltd., Huntington, UK) was used as a primary substrate. The FEP film was plasma treated with Ar plasma discharge, using a Balzers SCD 050 device (Bal-Tec AG, Lichtenstein). The gas purity was 99.997% with a gas pressure of 10 Pa. The treatment parameters were as follows: plasma power 8 W and exposure time 240 s, with an electrode distance of 50 mm.

The polystyrene was purchased from Goodfellow as oriented polystyrene foils with a thickness of 50 μm (PS, 1.05 g cm^−3^, Tg~100 °C). Polystyrene solutions were homogenized in an ultrasonic bath XUBA 1 (Grant Instruments (Cambridge) Ltd., Cambridgeshire United Kingdom) for 300 s. PS microstructures on the FEP were constructed by the improved phase-separation process using the dip-coating method; the PS solution of chloroform and methanol (100 mL, 90:10 by volume) was prepared and mixed with 2 g of PS until a homogeneous solution was produced. Briefly, IPS is a simple two-step method, where, in the first step, a polymer layer is applied to a solid substrate, and in the second step, the substrate is immersed in a mixture of two solvents: “good” (chloroform) and “bad” (methanol). After extracting the substrate from the binary mixture of solvents, they evaporated into the air. Because chloroform is more volatile, it evaporates rapidly and methanol droplets accumulate on the surface of the polymer. At the same time, based on the BF principle, the rapid evaporation of chloroform cools the polymer surface, and the high relative humidity condensed air water vapor into a polymer phase rich in methanol. Water causes an acceleration of droplet growth and an increase in droplet surface tension. This is essential for the conformational stability of methanol droplets, when the droplets are tightly packed due to capillary forces, and thus form a porous structure. The effect of humidity on the formation of HCP was confirmed by Bui in an experiment conducted in a dry environment [4].

**Figure 1:**
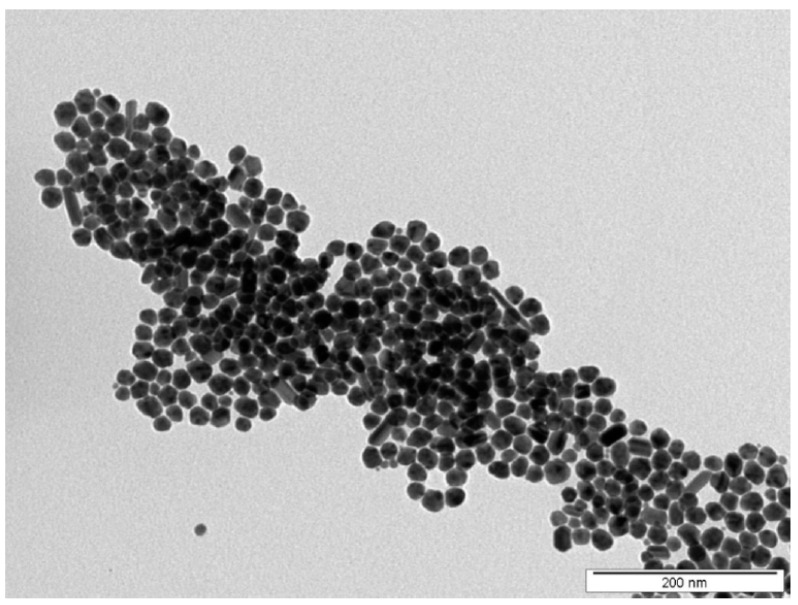
TEM image of synthesized AgNPs used in the immobilization process.

Silver nanoparticles were prepared by the electrochemical dissolution of two Ag electrodes in sodium citrate electrolyte according to the procedure described in [24]. Following the synthesis, the concentration of an AgNPs colloid was determined by AAS. For further experiments, the concentration of AgNPs was set to 30 mg L^−1^ by adding a 1 mM solution of sodium citrate. Prior to the immobilization process, AgNPs colloids are characterized by TEM (Figure 1).

The TEM characterization of AgNPs colloids was performed using JEOL JEM-1010 (JEOL Ltd., Akishima, Japan) operated at 80 kV. The particle size was measured using the TEM micrographs and calculated by considering at least 500 particles using the AnalysSIS 2.0 software. The average size of spherical AgNPs was approximately 20 nm. The samples for TEM were centrifuged, and NPs were transferred into distilled water. A drop of colloidal solution was placed on a copper grid coated with a thin amorphous carbon film on filter paper. The samples were air-dried and kept under vacuum in a desiccator before being placed on a specimen holder.

**Figure 2:**
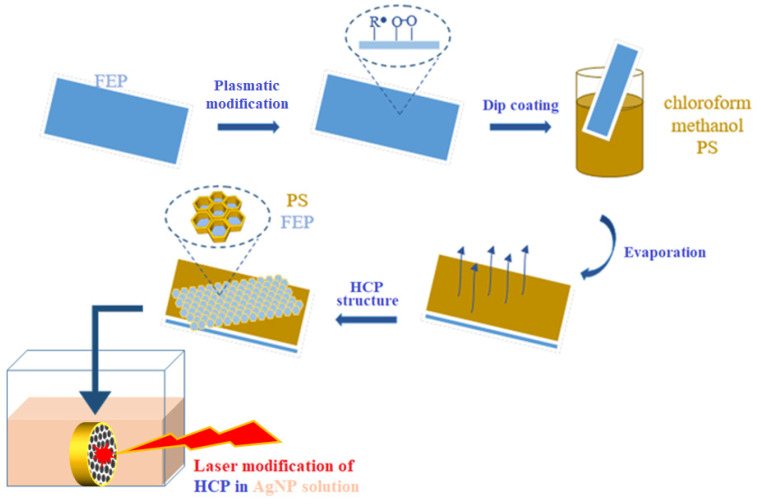
Scheme of the preparation of a honeycomb pattern immobilized with Ag nanoparticles.

The immobilization process (see Figure 2) was conducted using a KrF excimer laser (COMPex Pro 50F, Coherent, Inc., Silicon Valley, CA, USA; wavelength: 248 nm; pulse duration: 20–40 ns; repetition rate: 10 Hz; 6000 pulses). Polystyrene foil with a honeycomb pattern (preparation method as discussed in Section 3) was centered in the spectroscopic cuvette (Starna Scientific Ltd., Ilford, UK, type 3/Q/100) and charged with the AgNPs solution. In this set-up, PS foil was irradiated by 6000 laser pulses at a fluence of 10 mJ cm^−2^ through a linear polarizer (UV-grade fused silica prism, model PBSO-248-100).

### 2.2. Analytical Methods

The study of material surface morphology was performed using atomic force microscope Dimension ICON (Bruker Corp. Billerica, MA, USA). ScanAsyst mode in air was applied with a silicon tip on Nitride Lever SCANASYST-AIR and spring constant of 0.4 N.m^−1^. The acquired data were processed using NanoScope Analysis software 1.80.

The concentration of Ag in the colloidal solutions was determined by atomic absorption spectrometry (AAS) with a flame atomization technique. Measurements were obtained using a Varian AA880 device (Varian Inc., Palo Alto, CA, USA). The typical uncertainty of concentration determined by this method was less than 3%.

The surface visualization of the samples with immobilized AgNPs was determined using a high-resolution FEGSEM microscope MAIA3 (TESCAN, Brno, Czech Republic) equipped with detectors for secondary and backscattered electrons. Measurements were obtained in high-resolution mode at an accelerating voltage of 3 kV.

## 3. Results

### 3.1. Surface Morphology—Honeycomb Pattern (HCP)

An FEP polymer was used as a substrate for the preparation of the scaffold. The foil was modified with argon plasma at different powers (3 and 8 W) and an exposure time of 240 s. Plasma treatment was performed to strengthen the attachment of the polystyrene layer and to alter the wettability for the subsequent step of the improved phase separation of the polystyrene. Based on the improved phase separation, HCPs were successfully formed (as stated in scheme in Figure 2) on all modified surfaces.

**Figure 3:**
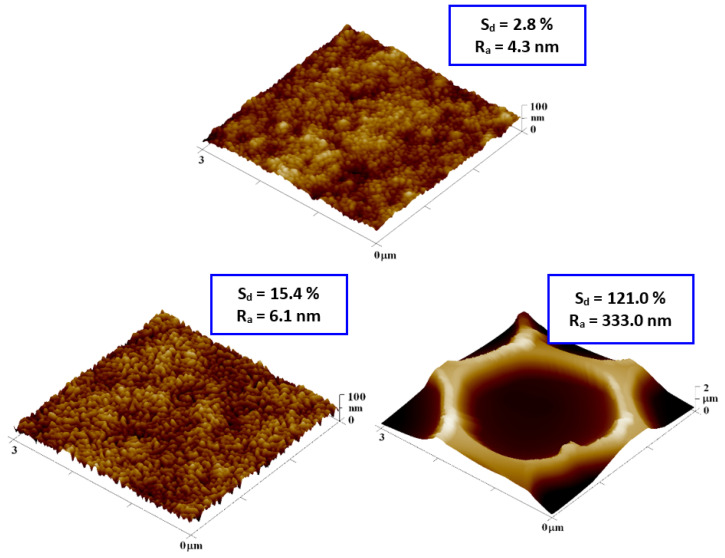
AFM scans (3 × 3 µm^2^) of plasma-modified FEP (3 W/8 W/240 s) and subsequently coated with PS HCP (8 W/240 s + 2 g PS). R_a_ represents the average of the deviations from the center plane of the sample and S_d_ represents the difference from the basic area.

**Figure 4:**
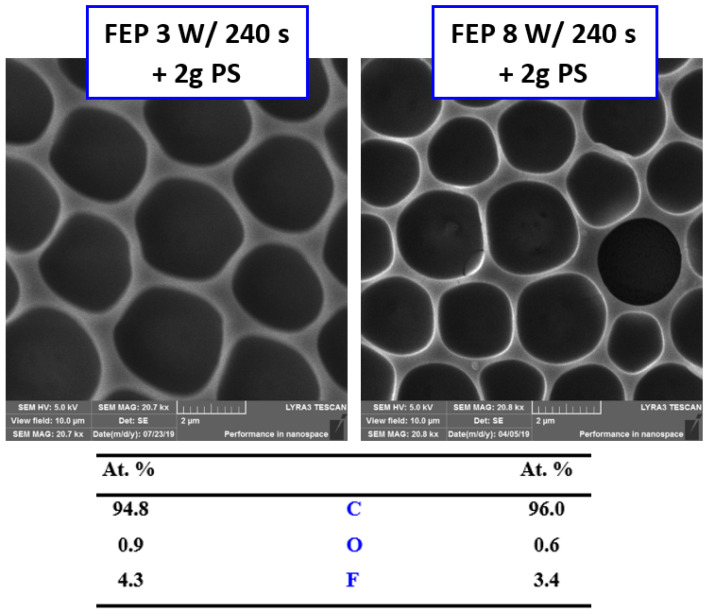
SEM scans (10 × 10 µm^2^) of plasma-modified FEP with PS HCP treated at different plasma discharge values (3 and 8 W); corresponding EDS graph and table of element concentrations on the surface.

Figure 3 presents the surface morphology of the FEP foil prior to and following the preparation of the honeycomb pattern. The creation of honeycomb patterns is described in the IPS method, which is based on the presence of methanol in a binary mixture of organic solvents. Methanol substitutes for a humid environment, stabilizes water droplets, and guarantees the creation of a regular pattern. Plasma treatment slightly increased the surface roughness; thus, the surface chemistry was significantly altered [32]. The enlarged image of the plasma-modified sample presented a wrinkled structure on the FEP surface. Images of the samples treated at a high power (8 W) and for a long exposure time (240 s) were selected for further experimentation. The formation of the honeycomb film significantly increased in surface area (121.0%) and roughness R_a_ (333.0 nm), as expected. The pores were circular and regularly arranged. The effect of different plasma-power values (3 and 8 W) at the same modification time (240 s) on the pore size and shape and the corresponding chemical surface compositions are presented in Figure 4. Circular-shaped pores were created. The results of the elemental analysis of these patterns are presented in Figure 4. The values listed in the table show that the chemical composition does not change when using different plasma discharges, and oxygen concentration significantly increases when compared to pristine FEP [24].

**Figure 5:**
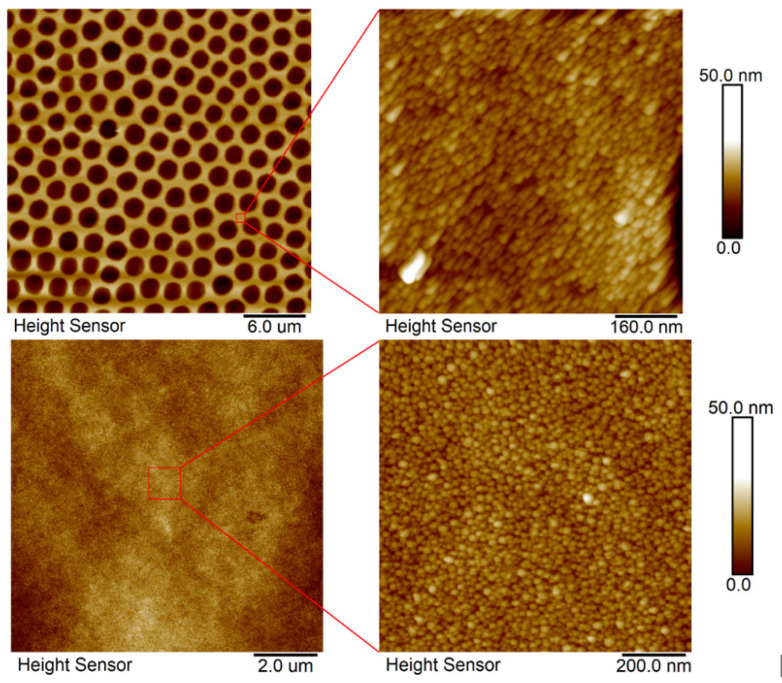
AFM scans of honeycomb pattern immobilized with Ag nanoparticles from the solution (upper line), 30 micron scan and 500 nm detail scans are presented. Morphology of polystyrene foil without the honeycomb pattern, which was exposed to the same conditions, is introduced in the bottom line of this Figure: 10 micron scans on the left-hand side.

As stated above, the preparation of the primary honeycomb polystyrene layer was based on an improved phase-separation technique in combination with dip coating; the process is described in detail in [7]. We aimed to immobilize noble metal nanoparticles into this structure, on the basis of the excimer exposure of the sample, which was immersed in the nanoparticle solution. The immobilization can be performed also on polymer without the honeycomb pattern. The key element is the aromatic core, so that it may interact with the applied excimer wavelength (248), the excimer exposure guides the immobilization process. One has to point out that the plasma treatment is a crucial step for primary surface modification (construction of honeycombs prior to further excimer exposure), which induces surface physico-chemical changes in FEP, changes its morphology and wettability properties, while the contact angle decreases [7,8,9]. The morphology of laser-immobilized samples of polystyrene honeycomb patterns is presented in Figure 5 (first line) for two different scanning areas. For the purpose of comparison, the results obtained from the immobilized polystyrene pristine samples are introduced in the bottom line of Figure 5. The immobilization process is successful, and the honeycomb structure is covered with globular noble metal nanostructures, as it is evident from the images on the right-hand side of Figure 5.

### 3.2. Surface Chemistry and SEM Analysis

**Figure 6:**
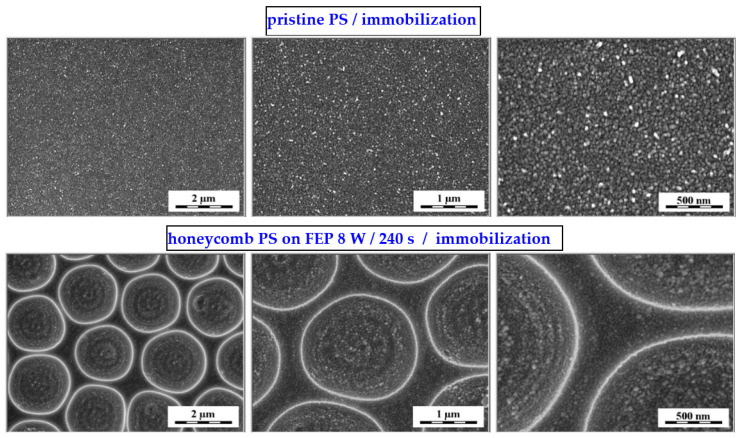
SEM scans of three different scanning areas of polystyrene honeycomb patterns immobilized with Ag nanoparticles from the solution (bottom line) and pristine polystyrene foil, which was exposed to the same conditions.

**Figure 7:**
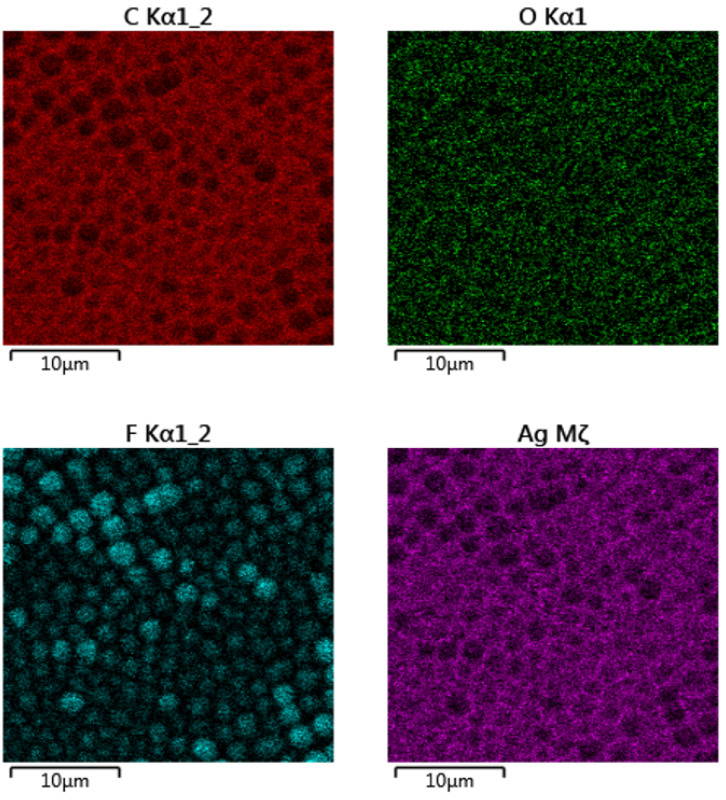
EDS mapping channels for carbon, oxygen, fluorine, and silver of the immobilized honeycomb polystyrene pattern; 30 micron scans are presented.

The SEM analysis revealed the successful surface immobilization of silver nanoparticles onto the honeycomb pattern (Figure 6). As introduced in the first line of Figure 6, the principle of immobilization was used primarily on the pristine polystyrene (not the honeycombs), and thus different quantities of silver may have been immobilized on the polystyrene surface. This approach was extended to the honeycomb pattern, where the immobilization process led to a uniform formation of Ag nanoclusters on the surface layer of the honeycomb units. The immobilized nanoparticles on the polystyrene pattern are clearly visible in Figure 6 (second line), a detailed image of the immobilized nanoparticles on the hexagonal pattern on the right-hand side of the image. In the figure, it is evident that immobilization occurs homogeneously over the large area of the constructed hexagonal pattern, with the morphology of immobilized nanoparticles being similar to that of pristine PS. The formation of the enriched layer and its homogeneity is well-documented in Figure 7. From the elemental mapping, it is clear that the honeycomb pattern is preserved over a large area. The most important point is that the immobilization is homogeneously realized on the round areas of the honeycomb pattern, as well as on the edges of the structure (bottom-right side of map). Additionally, the oxygen concentration is maintained over the surface area.

**Figure 8:**
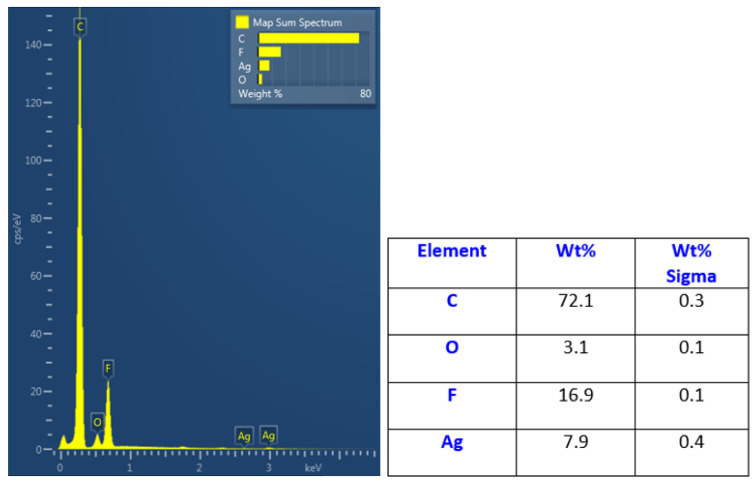
EDS spectrum of immobilized honeycomb pattern with silver acquired from a 30 × 30 μm^2^ area and corresponding elemental concentrations in the bottom part of the figure.

**Figure 9:**
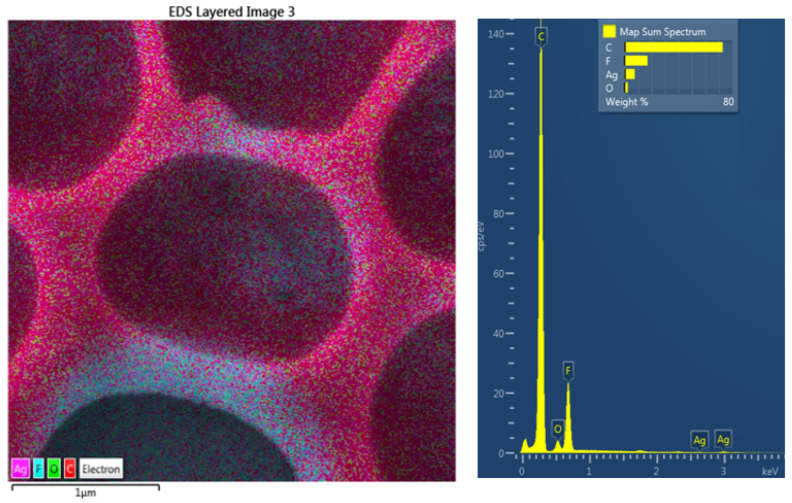
EDS mapping image superposed on SEM scan (3 × 3 micron square, on the left-hand side) of immobilized honeycomb pattern with silver, with EDS spectrum and corresponding elemental concentrations in this area.

The elemental composition of the immobilized honeycomb structure is presented in Figure 8. It is evident that the immobilization led to a significant increase in silver concentration in the upper layers of the honeycomb structure. The EDS method is able to provide information from several hundreds of nm; therefore, it can be expected that the surface is even more enhanced by silver nanoparticles. They also diffuse during the immobilization process into the bulk material, thus forming an Ag–polymer composite, as previously described by Siegel et al. [24]. The 8 wt.% concentration confirmed the high immobilization ratio and thus the possibility to apply the abovementioned technique for this type of polymer and polymeric structures. Moreover, we also applied EDS mapping to one honeycomb unit, as is presented in Figure 9. We confirmed the homogeneous elemental distribution of Ag and the concentration of silver to be approx. 8 wt.%, which is the same value as for large-scale mapping presented in Figure 7. It is obvious that the signal produced by the immobilized silver nanoparticles is collected from the area almost homogeneously (purple), which confirms the success of the immobilization process. Some minor areas presented less uniform or homogeneous immobilization, but the whole surface was still enhanced by silver.

## 4. Conclusions

In this experiment, we suggested a very simple and robust technique for large-scale patterning with polystyrene honeycomb units followed by silver immobilization from a silver nanoparticle solution supported by excimer laser treatment. The polystyrene honeycombs were successfully constructed of perfluorinated substrate, FEP, activated with argon plasma. The immobilization obtained from the solution was then successfully applied to the polystyrene HCP structure. This technique can be extended to other aromatic polymers, such as polycarbonate, which are able to interact with an excimer laser wavelength. The application of surface-immobilized polymers can be predominantly observed in SERS analysis, but also as constructs for soft and hard tissue engineering and in the construction of antibacterial surfaces.

## Data Availability

The data are contained within the article.
